# Motor Neuron Disease with Guillain-Barré Syndrome? Motor Band Sign with Anti-GQ1b Antibodies

**DOI:** 10.3390/diagnostics16050676

**Published:** 2026-02-26

**Authors:** Koji Hayashi, Asuka Suzuki, Mamiko Sato, Yuka Nakaya, Taibo Uchida, Tomohisa Yamaguchi, Toyoaki Miura, Hiromi Hayashi, Kouji Hayashi, Yasutaka Kobayashi

**Affiliations:** 1 Department of Rehabilitation Medicine, Fukui General Hospital, 55-16-1 Egami-cho, Fukui 910-8561, Japansatomoko@f-gh.jp (M.S.);; 2 Graduate School of Health Science, Fukui Health Science University, 55-13-1 Egami, Fukui 910-3190, Japanyasutaka_k@fukui-hsu.ac.jp (Y.K.); 3 Department of Neurology, University of Fukui Hospital, 23-3 Matsuoka Shimoaizuki, Eiheiji-cho, Yoshida-gun, Fukui 910-1193, Japan

**Keywords:** motor neuron disease, gangliosides, amyotrophic lateral sclerosis, Parkinsonism, GQ1b ganglioside, 9-O-acetyl-GD1b ganglioside, dementia

## Abstract

A 79-year-old former marathoner, with memory impairment since age 78, developed increasing stumbling and progressively worsening waddling gait. Three months after gait disturbance onset, she noted mild dysphagia. With declining walking distance and endurance, she presented to our hospital six months after onset, exhibiting frontal signs, Parkinsonism with marked trunk rigidity, and hyperreflexia of the jaw and limbs. L-dopa challenge tests showed no improvement. At seven months post-onset, she had difficulty rising. By nine months, she relied on a walker, and speech disturbance appeared. At 10–11 months, both dysarthria and dysphagia rapidly worsened, she became bed-ridden, and upper limb weakness developed (though she could still use chopsticks). Neurological examination at one year revealed severe dysarthria/dysphagia, four extremity fasciculations and muscle weakness (grade 2 in upper limbs, grade 1 in lower limbs), trunk-dominant rigidity, and hyperreflexia in the jaw and limbs. Brain MRI, specifically susceptibility-weighted imaging, revealed motor band signs. Cerebrospinal fluid study revealed albuminocytological dissociation. Needle electromyography revealed acute denervation and chronic reinnervation in the cranial nerve, cervical, and lumbar areas, which was suggestive of motor neuron disease (MND). Serum anti-GQ1b antibodies were detected. Immunotherapy was followed by mild improvement, which might suggest a reversible component, although definitive pathological overlap remains unconfirmed. This case highlights a diagnostic challenge where an acute immune-mediated neuropathy could potentially be superimposed on a chronic neurodegenerative process. Anti-GQ1b antibodies should be interpreted with caution, as they may reflect either a true clinicopathological overlap with Guillain-Barré syndrome or a secondary phenomenon (epiphenomenon) related to the primary neurodegenerative process.

**Figure 1:**
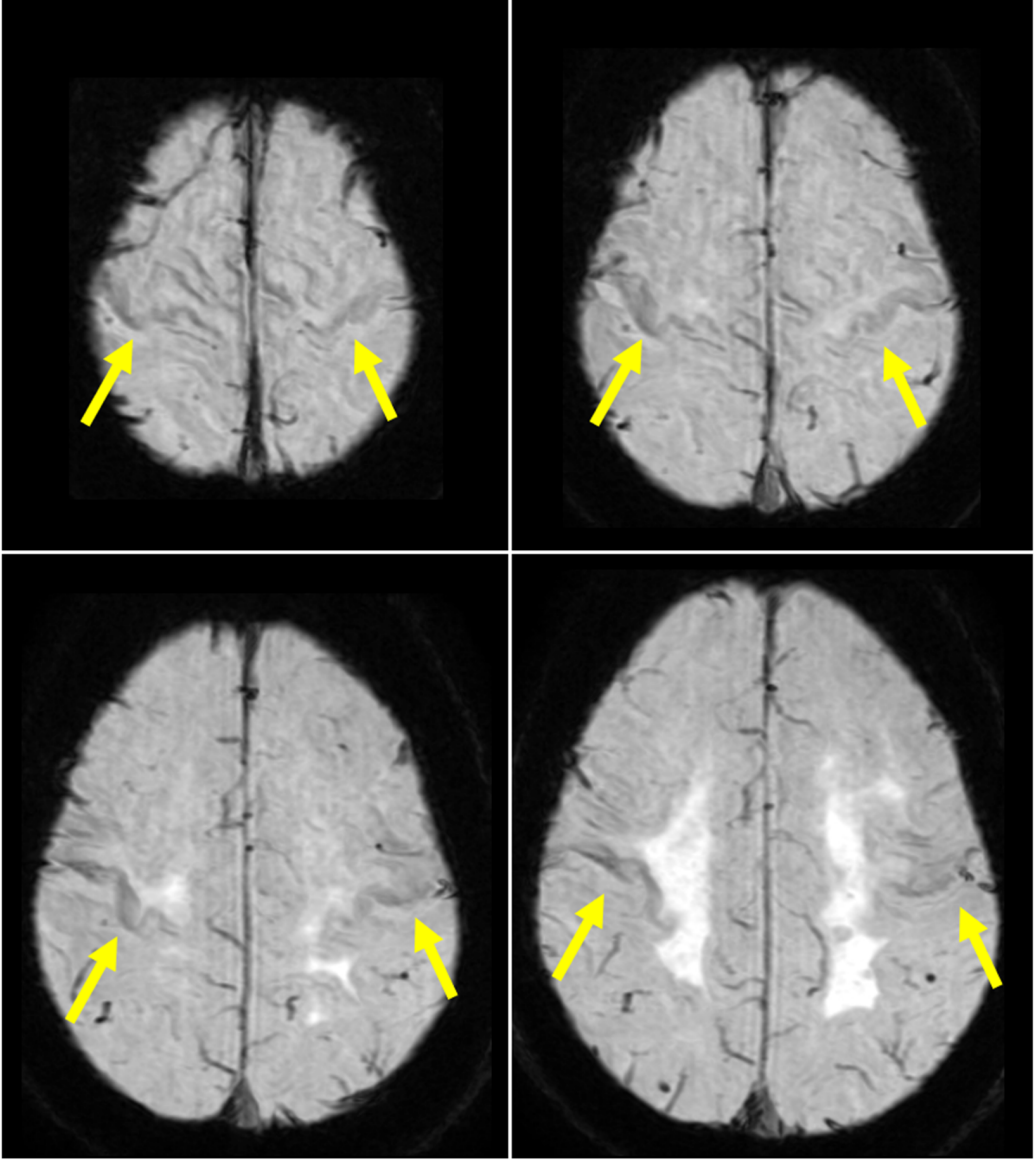
**Brain magnetic resonance imaging (MRI) on susceptibility-weighted imaging (SWI) results.** SWI showing hypointensities in the bilateral precentral gyrus (arrows), consistent with the motor band sign (MBS) (at 1.5 Tesla using a 3D-gradient echo; Revolution^TM^ Maxima; GE Healthcare, Chicago, IL, USA). MBS, also known as the “black ribbon sign,” is a distinctive imaging characteristic that manifests as a curved region of reduced signal intensity within the precentral gyrus, more precisely involving the primary motor cortex (M1) [1,2,3]. This finding is primarily seen in patients with motor neuron diseases (MND), notably Amyotrophic Lateral Sclerosis (ALS) and Primary Lateral Sclerosis (PLS), and serves as an indicator of upper motor neuron (UMN) dysfunction [1,2,3,4]. The hypointensity defining MBS is primarily attributed to neurodegenerative iron accumulation within the primary motor cortex [1,2,4]. Postmortem histopathological studies have confirmed that these iron deposits occur preferentially within microglia located in the deep layers of the primary motor cortex [1,2]. This accumulation is believed to be a hallmark of neuroinflammatory processes, where microglial activation leads to the release of proinflammatory cytokines and reactive oxygen species, ultimately contributing to the degeneration of corticospinal neurons [1,2]. Although the exact mechanisms of iron overload are not yet fully understood, they are hypothesized to involve a combination of oxidative stress, mitochondrial dysfunction, abnormal iron metabolism, and macrophage infiltration [2]. Among several MRI sequences, SWI is the most sensitive routine sequence for detecting MBS, as it utilizes both magnitude and phase data to enhance contrast from local susceptibility changes, such as iron content [1,2,4]. In a clinical setting, MBS serves as a highly specific diagnostic tool for identifying ALS, demonstrating a specificity of 91.1% versus disease mimics and 96.8% against healthy controls [1]. While its diagnostic utility is tempered by a relatively limited sensitivity of approximately 59.6%, the sign remains invaluable for distinguishing ALS from other neurodegenerative conditions and common “mimics,” such as spinal stenosis, Parkinson’s disease, and polyneuropathy [1,2]. Nevertheless, while MBS is highly suggestive of MND, it is not strictly exclusive to ALS [2,3]. It has been documented in other neurodegenerative conditions, most notably Huntington’s disease and its phenocopy, spinocerebellar ataxia type 17 (SCA17), as well as in Alzheimer’s disease and Parkinson’s disease [2,3]. Additionally, the sign is occasionally observed in healthy individuals or those with acquired disorders, such as stroke and progressive multifocal leukoencephalopathy [2]. This case is particularly notable for the co-occurrence of the MBS (as well as MND features) with albuminocytological dissociation in the cerebrospinal fluid and serum anti-GQ1b antibody positivity—features typically suggestive of an immune-mediated neuropathy such as Guillain-Barré syndrome (GBS). While this clinical presentation is intriguing, the presence of anti-GQ1b antibodies should be interpreted with caution; it may represent a true clinicopathological overlap or, alternatively, an associated secondary phenomenon (epiphenomenon) related to the primary neurodegenerative process. Although anti-GQ1b antibodies in ALS are rare and sometimes considered an epiphenomenon of motor neuron destruction [5], the rapid clinical deterioration and subsequent mild but clear improvement following immunotherapy in our case could potentially suggest an associated reversible immune-mediated component.

**Figure 2:**
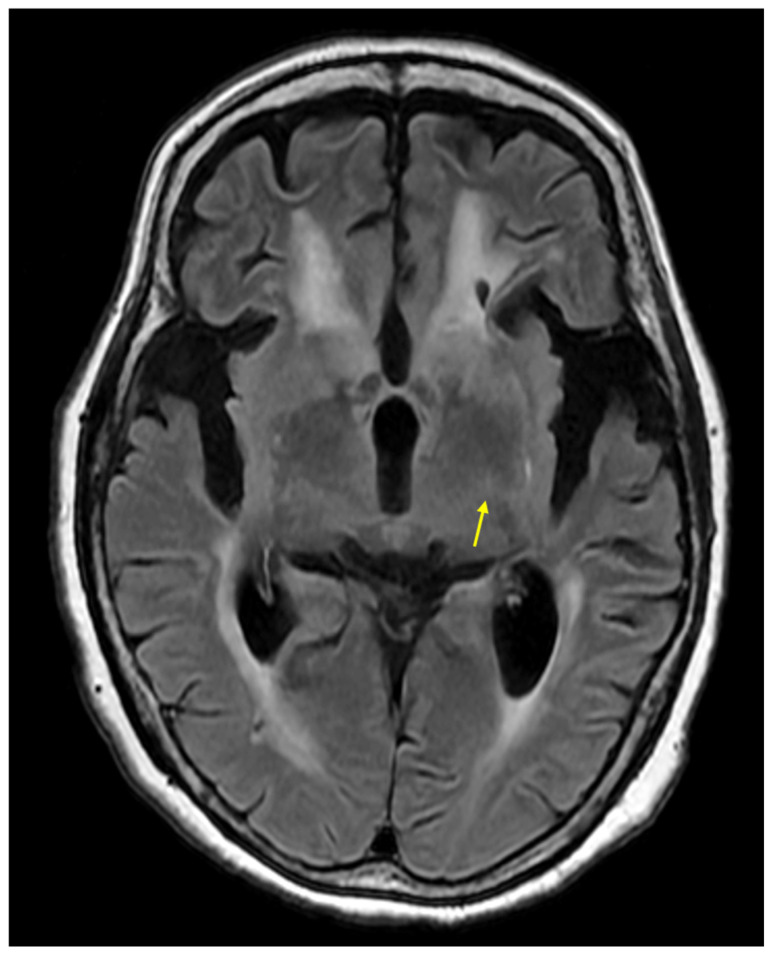
**Brain MRI on T2-fluid attenuated inversion recovery (T2-FLAIR) results.** In our case, a slight hyperintensity was noted in the left posterior limb of the internal capsule (PLIC) near the left basal ganglia (arrow). Hyperintensity in the PLIC on T2-FLAIR images is widely recognized as a hallmark of corticospinal tract (CST) degeneration in ALS [6,7]. Previous studies have reported that bilateral CST hyperintensity is observed in approximately 40% (median value) of ALS patients with predominant UMN signs on T2, FLAIR, or proton density-weighted images [6,7]. On the other hand, its diagnostic utility remains a challenge, as similar hyperintensities on FLAIR images are frequently observed even in healthy control groups, leading to concerns regarding its specificity [6,7]. Furthermore, the absence of CST finding does not rule out ALS [8]. Moreover, advanced techniques like diffusion tensor imaging (DTI) are recognized for providing more detailed quantitative data of upper motor neuron degeneration in ALS [8]. Specifically, DTI-derived metrics such as fractional anisotropy can serve as sensitive indicators of microstructural axonal loss and white matter integrity, often detecting changes even when conventional MRI findings are subtle [6,9]. We acknowledge that the lack of DTI measurements is a limitation for further quantitative assessment in our case.

**Figure 3:**
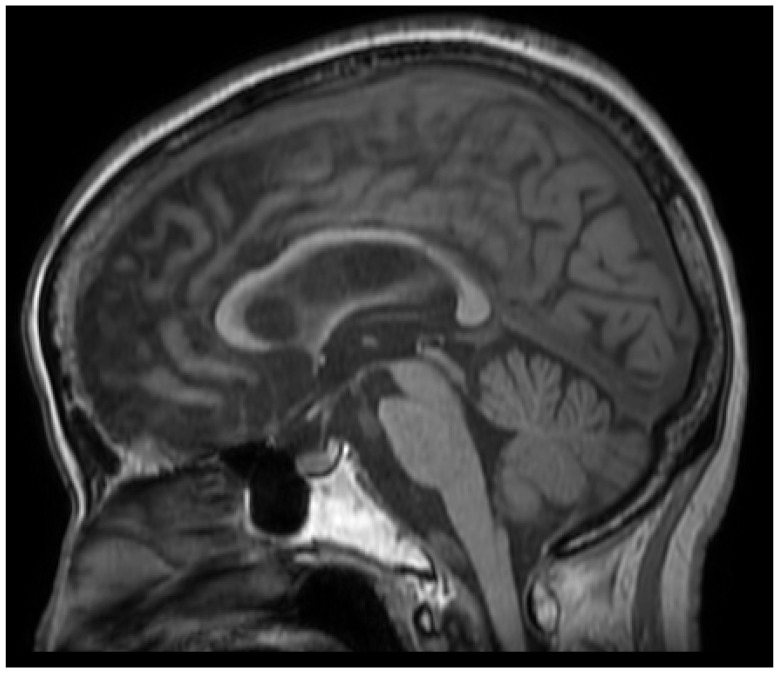
**Brain MRI on T1-weighted imaging results.** No atrophy of the brainstem or cerebellum, including the midbrain tegmentum, was observed. To objectively evaluate the clinical Parkinsonism as well as hyperreflexia observed in this case, the magnetic resonance Parkinsonism index (MRPI) was calculated. The MRPI is a robust quantitative imaging marker that is used to differentiate progressive supranuclear palsy (PSP) from Parkinson’s disease and other Parkinsonian syndromes by measuring the area of the pons and midbrain, and the width of the middle and superior cerebellar peduncles [10,11]. While an MRPI value of ≥12.6 is highly sensitive and specific for a diagnosis of PSP [10,11], the MRPI in our patient was 1.79. This significantly low value, along with the preserved midbrain and superior cerebellar peduncles, helps distinguish the patient’s Parkinsonian features from the structural changes typical of classic PSP, supporting the interpretation that these clinical signs may represent a distinct phenotype or an associated phenomenon rather than primary midbrain degeneration [10,11].

**Figure 4:**
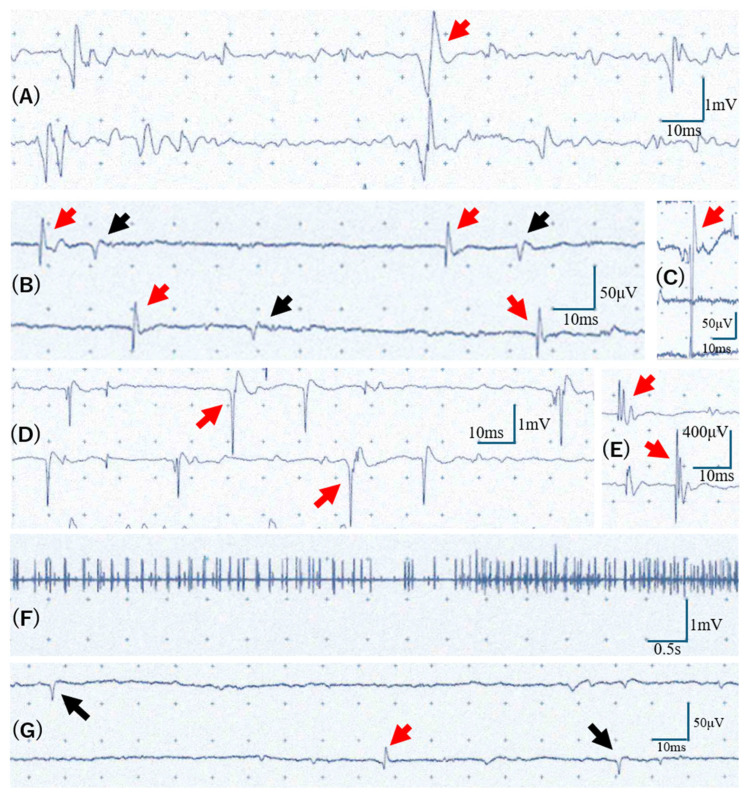
**Needle electromyography (nEMG) findings.** (**A**) Tongue: Large-amplitude motor unit potentials (MUPs) exceeding 2 mV were noted during weak voluntary contraction (arrow); resting activity and interference could not be assessed due to the patient’s cognitive decline. (**B**) Brachioradialis (at rest): Fibrillation potentials (red arrows) and positive sharp waves (black arrows) were observed. (**C**) Brachioradialis (at rest): Fasciculation potentials were also noted (arrow). (**D**) Biceps brachii: Large-amplitude MUPs exceeding 2 mV were demonstrated during weak voluntary contraction (arrows). (**E**) Biceps brachii: Additionally, polyphasic MUPs were observed (arrow) during voluntary contraction. (**F**) Extensor digitorum communis: A poor interference pattern with reduced MUP recruitment was seen during maximal effort. (**G**) Tibialis anterior (at rest): Fibrillation potentials (red arrow) and positive sharp waves (black arrows) were observed. While only a representative portion of the waveforms is depicted here, these potentials appeared repeatedly at regular intervals. Profound weakness in the lower limbs precluded the assessment of voluntary contractions and interference patterns. Based on the Awaji revision of the El Escorial criteria [12], this case can be classified as “Clinically Definite” ALS, as it demonstrates evidence of both UMN and lower motor neuron (LMN) involvement across three distinct regions. In the bulbar region, UMN involvement was evidenced by hyperreflexia of the jaw-jerk reflex, while needle electromyography (nEMG) confirmed LMN dysfunction through large-amplitude motor unit potentials (MUPs) exceeding 2 mV in the tongue. The cervical region exhibited clinical UMN and LMN signs, including hyperreflexia, muscle atrophy, and fasciculations. Additionally, nEMG revealed active denervation (fibrillation potentials and positive sharp waves) and chronic reinnervation (large-amplitude polyphasic MUPs with reduced recruitment) in the upper limb muscles, including the brachioradialis. In the lumbosacral region, UMN dysfunction was supported by hyperreflexia, while the 1-year course of progressive leg weakness and nEMG findings of fibrillation potentials and positive sharp waves in the tibialis anterior confirmed LMN involvement. By integrating these clinical and electrophysiological findings—specifically acknowledging that nEMG abnormalities are equivalent to clinical LMN signs and that fasciculation potentials serve as evidence of acute denervation under the Awaji criteria—this patient meets the diagnostic requirements for a definitive diagnosis of ALS across the bulbar, cervical, and lumbosacral regions. This case highlights a diagnostic challenge in which an acute immune-mediated neuropathy (GBS PCB-variant) may have been superimposed on a chronic neurodegenerative process (ALS). The Awaji criteria allowed for a ‘Clinically Definite’ diagnosis by integrating clinical and electrophysiological findings; however, clinicians should remain alert to atypical rapid progression and anti-ganglioside positivity as factors that might prompt consideration of treatable overlap syndromes. Beyond the MND, the patient exhibited Parkinsonism. Although her ancestral origin remains unknown, the clinical presentation resembled the ALS–Parkinsonism–dementia complex (ALS/PDC) endemic to the Kii Peninsula of Japan [13]. As the patient has requested a posthumous body donation for medical education, further details of her pathology may be revealed in the future.

## Data Availability

The data presented in this study is available on request from the corresponding author. Due to patient privacy and ethical considerations, the data is not publicly accessible.
